# Effects of Time-Restricted Eating (Early and Late) Combined with Energy Restriction vs. Energy Restriction Alone on the Gut Microbiome in Adults with Obesity

**DOI:** 10.3390/nu17142284

**Published:** 2025-07-10

**Authors:** Bernarda Habe, Tanja Črešnovar, Matjaž Hladnik, Jure Pražnikar, Saša Kenig, Dunja Bandelj, Nina Mohorko, Ana Petelin, Zala Jenko Pražnikar

**Affiliations:** 1 Faculty of Health Science, University of Primorska, Polje 42, 6310 Izola, Slovenia; bernarda.habe@fvz.upr.si (B.H.); tanja.cresnovar@fvz.upr.si (T.Č.); sasa.kenig@fvz.upr.si (S.K.); nina.mohorko@upr.si (N.M.); ana.petelin@upr.si (A.P.); 2 Faculty of Mathematics, Natural Sciences and Information Technologies, University of Primorska, Glagoljaška 8, 6000 Koper, Slovenia; matjaz.hladnik@famnit.upr.si (M.H.); jure.praznikar@upr.si (J.P.); dunja.bandelj@famnit.upr.si (D.B.); 3 Andrej Marušič Institute, University of Primorska, Muzejski trg 2, 6000 Koper, Slovenia

**Keywords:** eating window, energy restriction, gut microbiota, alpha and beta diversity, metabolic health, obesity

## Abstract

**Background:** Early time-restricted eating combined with energy restriction (eTRE + ER) has been shown to reduce fat mass, diastolic blood pressure (DBP) and fasting glucose more effectively than late TRE with energy restriction (lTRE + ER) or energy restriction (ER) alone. Given the gut microbiome’s sensitivity to circadian rhythms, we examined whether adding TRE, particularly eTRE, to ER alters gut microbiota composition beyond ER alone, and whether such effects persist during follow-up. **Methods:** We analysed anthropometric, biochemical and gut microbiome data from 76 participants at baseline and after a 3-month intervention (eTRE + ER: *n* = 33; lTRE + ER: *n* = 23; ER: *n* = 20). Follow-up microbiome data 6-months after the end of intervention were available for 43 participants. Gut microbiota composition was assessed via 16S rRNA gene sequencing of stool samples. **Results:** No significant between-group differences in beta diversity were observed over time. However, changes in alpha diversity differed significantly across groups at the end of the intervention (Shannon: F = 5.72, *p* < 0.001; Simpson: F = 6.72, *p* < 0.001; Richness: F = 3.99, *p* = 0.01) and at follow-up (Richness: F = 3.77, *p* = 0.02). lTRE + ER led to the greatest reductions in diversity post intervention, while ER was least favourable during follow-up. Although no significant between-group differences were observed at the phylum level either at the end of the intervention or during follow-up, only the eTRE + ER group exhibited a significant decrease in Bacillota and an increase in Bacteroidota during follow-up. At the genus level, differential abundance analysis revealed significant shifts in taxa such as *Faecalibacterium*, *Subdoligranulum*, and other genera within the *Ruminococcaceae* and *Oscillospiraceae* families. In the eTRE + ER, *Faecalibacterium* and *Subdoligranulum* increased, while in other groups decreased. Notably, the changes in *Faecalibacterium* were negatively correlated with fasting glucose, while the increase in *Subdoligranulum* was inversely associated with DBP; however, both associations were weak in strength. **Conclusions**: eTRE + ER may promote beneficial, lasting shifts in the gut microbiome associated with improved metabolic outcomes. These results support further research into personalized TRE strategies for treatment of obesity.

## 1. Introduction

Obesity is a global epidemic that significantly increases the risk of type 2 diabetes mellitus (T2DM), cardiovascular diseases, and certain cancers [1]. Among the various factors contributing to obesity, nutrition plays a crucial role, making dietary interventions a key strategy for weight management [2]. One such intervention, time-restricted eating (TRE), is an intermittent-fasting approach that limits the daily eating window. TRE has been shown to be an effective weight-loss strategy, reducing body mass, body fat, and metabolic risk factors in humans [3,4]. Given that TRE reduces overall dietary intake and alters the availability of nutrients in the distal colon during fasting periods, it is plausible to hypothesize that it may influence the composition and dynamics of the gut microbiome.

The gut microbiota plays a vital role in human health, contributing to vitamin and essential amino acid biosynthesis and producing key metabolic by-products such as short-chain fatty acids (SCFAs), including butyrate, propionate, and acetate. These SCFAs serve as major energy sources for intestinal epithelial cells and help strengthen the mucosal barrier [5]. Research suggests that gut microbiota also plays a role in obesity development [6,7]. Indeed, individuals with obesity often exhibit altered gut microbiota composition and function [8]. Specifically, lower microbial diversity and richness, along with a higher Bacillota-to-Bacteroidota ratio, have been associated with obesity [9,10], although the role of Bacillota remains unclear. Notably, interventions such as faecal microbiota transplantation from lean donors and supplementation with beneficial microbes like *Akkermansia muciniphila* have demonstrated potential in reducing body mass and improving metabolic parameters related to obesity [11]. Diet is known to modulate gut microbiota composition in both humans and animals [12,13].

It is known that the gut microbiome follows cyclical diurnal rhythms [14]. In rodents, bacterial populations of the Bacteroidota and Verrucomicrobia phyla peak during fasting periods and decrease as feeding time approaches, when Bacillota become dominant [14]. Meal timing may, therefore, influence gut microbiota composition, with implications for host health [5]. However, findings on the effects of TRE on the gut microbiome remain mixed. Li et al. [15] suggest that intermittent energy restriction (reduced daily caloric intake compared to daily energy requirement) can improve obesity outcomes by modulating gut microbiota composition in mice. Similarly, intermittent fasting, including TRE, has been linked to reduced overall gut bacterial populations and increased microbial diversity in healthy individuals [16,17]. Some available studies have reported that TRE is associated with increased levels of either *Akkermansia muciniphila*, *Bacteroides fragilis*, *Prevotellaceae, Bacteroidaceae*, or *Butyricicoccus pullicaecorum*, promoting greater gut microbial diversity [18,19,20,21]. Conversely, some studies have reported no significant differences in gut microbiota diversity or composition between TRE and unrestricted eating [22,23,24]. Animal studies in paediatric mice have even suggested that time-restricted feeding may disrupt microbiota–host interactions and lead to persistent gut microbiota impairments [25,26].

Due to the small sample sizes in previous studies, research findings remain inconsistent, and the relationship between TRE and gut microbiota composition requires further investigation. Also, it is not clear yet if gut microbial alterations are due to energy restriction or TRE. In our parent study, we found that early time-restricted eating (eTRE) combined with energy restriction (ER) was more effective in reducing fat mass percentage, diastolic blood pressure, and fasting glucose levels compared to late TRE (lTRE) with ER or ER alone after 12 weeks of intervention [4]. Building on these findings, we conducted a secondary analysis to assess whether adding TRE, particularly eTRE, to ER produces additional changes in the gut microbiome compared to ER alone and whether the effects are still present after 6 months’ follow-up.

## 2. Materials and Methods

### 2.1. Study Design

This study represents a secondary analysis of a three-arm, parallel, 3-month randomized clinical trial conducted between March and June 2023 at the University of Primorska, Faculty of Health Sciences. The detailed study protocol and primary findings have been previously published [4]. Ethical approval was obtained from the Slovenian National Medical Ethics Committee (No. 0120-557/2017/4; Ministry of Health, Republic of Slovenia), and the trial was registered on ClinicalTrials.gov (NCT05730231). Prior to participation, all subjects provided written informed consent.

### 2.2. Study Participants

Participants were recruited through online platforms, radio and television advertisements, social media, and local newspapers. The inclusion and exclusion criteria have been described in detail elsewhere [4]. In summary, eligible participants were aged 18 to 60 years with a body mass index (BMI) between 25 kg/m^2^ and 35 kg/m^2^, and met at least one criterion for metabolic syndrome. Exclusion criteria included the use of antihypertensive or cholesterol-lowering medications, an average daily eating window of less than 11 h, pregnancy or breastfeeding, smoking, active participation in a weight loss program, chronic illnesses (e.g., cardiovascular, gastrointestinal, oncological, or haematological diseases), diagnosed or past eating disorders, shift work, excessive alcohol consumption (>2 servings/day for men, >1 serving/day for women), or the use of dietary supplements, including probiotics, which could impact study outcomes. Moreover, individuals who had used antibiotics within three months before the study were excluded.

### 2.3. Intervention

The intervention protocol has been previously described [4]. Participants were randomized into three groups: (1) eTRE + ER, consuming all meals within an 8 h window from 8:00 to 16:00, (2) lTRE + ER, with an 8 h eating window from 12:00 to 20:00, and (3) ER, following a 12 h eating window from 8:00 to 20:00. Stratified randomization was performed using the open-source software MinimPy, 0.3 version (https://sourceforge.net/projects/minimpy, accessed on 2 February 2023). A total of 300 participants were screened, and 108 were enrolled. Ultimately, 93 participants completed the 3-month intervention, with gut microbiome data available for 76 individuals at baseline and after 12 weeks of specific intervention (eTRE + ER: *n* = 33; lTRE + ER: *n* = 23; ER: *n* = 20), while the gut microbiome data was available for only 43 participants after 6 months’ follow-up (eTRE + ER: *n* = 14; lTRE + ER: *n* = 14; ER: *n* = 15).

At baseline, all participants received individualized dietary guidance from a registered dietitian, aiming to reduce energy intake by approximately 2100 kJ (500 kcal) based on their resting metabolic rate and daily physical activity level. Macronutrient distribution was standardized across groups: approximately 50% carbohydrates, 20% protein, and 30% fat. Meals were evenly distributed: 30% of daily intake at breakfast, 40% at lunch, and 30% at dinner, with no snacks allowed between meals. Only water and unsweetened tea were permitted outside meal times. Monthly online motivational meetings with a dietitian were conducted to support adherence. Participants self-reported compliance with eating windows, energy intake, and meal frequency using a dietary calendar. Per-protocol analysis included participants who (a) adhered to their assigned TRE window (within a ±30 min tolerance per day), (b) (ER) consumed 3 meals per day and (c) completed all primary and secondary outcome assessments. At the introductory meeting, the participants were informed that if they did not follow the protocol for four consecutive days, they would be excluded from the study. These criteria were pre-defined in the study’s plan. Additionally, dietary intake was assessed via a 3-day food diary after two months, with data analysed using the Open Platform for Clinical Nutrition (OPEN) (http://opkp.si/, accessed on 1 March 2024).

### 2.4. Anthropometric and Biochemical Measurements

All assessments were conducted in the morning following 12–15 h of fasting. Body mass and composition were measured using a Tanita MC-980MA bioelectrical impedance analyser (Tanita Corporation, Arlington Heights, IL, USA) with GMON Pro-Tanita software (Version 3.2.9).

Venous blood samples were collected using 9 mL vacuum serum test tubes and 6 mL vacuum EDTA test tubes (Greiner Bio-One, Kremsmünster, Austria). Serum and plasma were separated by centrifugation (2000× *g* for 10 min), frozen, and stored at −80 °C for further analysis. Biochemical parameters, including fasting glucose, triacylglycerols (TG), total cholesterol, low-density lipoprotein (LDL) cholesterol, and high-density lipoprotein (HDL) cholesterol were analysed using a Cobas^®^ c111 analyser (Roche, Basel, Switzerland).

### 2.5. Gut Microbiome Analysis

For microbial analysis, fresh stool samples were collected at baseline (*n* = 76; eTRE + ER, *n* = 33; lTRE + ER, *n* = 23; ER, *n* = 20) after 12 weeks of the intervention (*n* = 76; eTRE + ER, *n* = 33; lTRE + ER, *n* = 23; ER, *n* = 20), and after follow-up (6 months after the end of the intervention; *n* = 43; eTRE + ER, *n* = 14; lTRE + ER, *n* = 14; ER, *n* = 15). The faecal samples were frozen immediately at −80 °C or briefly stored by participants in −20 °C freezers before being transported to the study centre within 12 h on ice, then stored at −80 °C until processing. DNA was extracted from frozen faecal samples (1–2 g) using the QIAamp DNA Stool Mini Kit (Qiagen N.V., Venlo, The Netherlands) following the manufacturer’s instructions. Amplification of V4 16S rRNA was performed with 515F and 806R primers. Each sample was amplified with a unique barcode in triplicate to reduce PCR bias. An equal amount of pooled triplicates of each sample was joined in a final library. The final library was then cleaned with Agencourt AMPure XP magnetic beads (Beckman Coulter, Brea, CA, USA) at a volume ratio of 0.7:1 beads: DNA. Sequencing was performed on Illumina platform by Novogene Co., Ltd. (Beijing, China), using 2 × 250 paired-end chemistry. Read orientation was normalized with Cutadapt version 5.0 (v5.0) (using the –revcomp option). The paired-end reads were then imported into QIIME2 (version 2024.10). Full-length amplicons were extracted, denoised, and amplicon sequence variants (ASVs) determined using the qiime cutadapt trim-paired and qiime dada2 denoise-paired methods. A Naïve Bayes classifier was trained on 99% identity SILVA 138 reference sequences and their taxonomy files, which were pre-formatted using the RESCRIPt plugin. The 515F/806R region was extracted with q2-feature-classifier plugin, and taxonomic assignments were performed using the classify-sklearn method. Reads were rarefied to 100,000 per sample to normalize sequencing depth across samples and avoid biases due to uneven read counts. This threshold was chosen based on rarefaction curves, which indicated that diversity metrics plateaued at this depth.

### 2.6. Statistical Analysis

The secondary analysis was conducted using SPSS version 29.0 (IBM Corp., Armonk, NY, USA) for clinical data and R packages (R package vegan (version 2.6-10)) for microbiome.

Normality of variables was assessed using the Shapiro–Wilk test. Baseline characteristics of the participants of all three groups (eTRE + ER, lTRE + ER and ER) are presented (as mean values and standard deviations). Differences in baseline characteristics between three groups were compared using ANOVA or the Kruskal–Wallis test, as appropriate. The treatment effect for anthropometric measures and laboratory parameters was evaluated using a general linear model, where *p* values have been adjusted by the Bonferroni corrections for multiple tests. Within-group changes were analysed using paired *t*-tests for normal distributions or paired Wilcoxon tests for non-normal distributions. A significance threshold of *p* < 0.05 was set for all statistical analyses. Data are reported as mean ± standard deviation (SD) or mean (95% confidence intervals).

The abundance of a taxon in a stool sample was indicated as the relative abundance, which was calculated by dividing the number of reads for a taxon by the total read counts of the sample. Alpha diversity indexes (Shannon index, Simpson index and richness index) were calculated using R. Bray–Curtis dissimilarity was used to evaluate functional diversity between samples. The effect of the interventions (eTRE + ER, lTRE + ER, ER) on beta diversity was tested using permutational multivariate analysis of variance (PERMANOVA) using the adonis and beta dispersion functions from the vegan R package with 999 permutations. The effect of the TRE intervention on alpha diversity (Shannon, Simpson, and number of taxa) was analysed using a general linear model, adjusted for baseline values. The effect of the TRE intervention on gut microbiota composition was analysed using two-tailed Wilcoxon rank-sum tests or Wilcoxon signed-rank tests for unpaired and paired samples, respectively, and adjusted by Benjamini–Hochberg false discovery rate (FDR) correction when multiple comparisons were applied. Spearman’s correlation analysis was performed to investigate the relationship between the abundance changes of significantly altered gut microbial at genus level and changes in clinical indices. All statistical analyses regarding gut microbiota were performed in R package-version 4.2.2 (R Core Team, 2022). All *p* values were two-tailed, and *p* < 0.05 was regarded as being statistically significant.

## 3. Results

### 3.1. Characteristics of Study Participants at Baseline, During the Intervention and After Follow-Up

Results of the primary and secondary metabolic outcomes have been reported elsewhere [4]. This secondary analysis focuses on a subset of participants from the original trial who had faeces samples available for analysis at baseline and after the 3-month intervention (*n* = 76), as well as after follow-up (*n* = 43).

At baseline, there were no significant differences among the eTRE + ER, lTRE + ER, and ER groups in terms of sex, age, body mass, or BMI, confirming that randomization was maintained in this analysis. Additionally, no significant differences were observed at baseline in fat mass percentage, muscle mass, resting energy expenditure, blood pressure, fasting glucose, triacylglycerol levels, or HDL cholesterol levels (Table 1). Eating windows, energy intake, and macronutrient intake were also similar across all three groups at the start of the intervention. However, significant differences were found at baseline for total cholesterol levels (*p* = 0.019). Specifically, participants in the lTRE + ER group had significantly higher total cholesterol levels than those in the eTRE + ER group.

Overall, during the intervention, participants in the TRE groups (eTRE + ER and lTRE + ER) successfully reduced their eating window and energy intake, whereas participants in the ER group reduced only their energy intake (Table 1). All three interventions led to weight loss and improvements in metabolic parameters during the intervention period. However, the eTRE + ER group experienced significantly greater reductions in fasting serum glucose (F = 3.19, *p* = 0.047, R^2^ = 0.08), and diastolic blood pressure (F = 3.65, *p* = 0.031, R^2^ = 0.09) compared to the ER and/or lTRE + ER groups (Table 1). In this sample, all three interventions had comparable effects on body mass, BMI, muscle mass, body fat mass, and lipid profile (Table 1). The present results, therefore, do not align with those of our parent study, which found that eTRE combined with ER was more effective in reducing fat mass percentage compared to lTRE + ER or ER alone after 12 weeks of intervention [4]. However, after adjusting for age, gender and baseline values, significant differences between groups emerged also for fat mass percentage (F = 3.32, *p* = 0.036, R^2^ = 0.09), with greater reductions observed in the eTRE + ER group than in the other two groups.

When only the follow-up sample (*n* = 43) was analysed, the between-group differences at baseline and post-intervention were similar to those observed in the full sample (*n* = 76). At baseline, there were no significant differences between groups. During the intervention, the same pattern was observed as in our parent study; the eTRE + ER group showed significantly greater reductions in fasting serum glucose (F = 2.98, *p* = 0.045, R^2^ = 0.13), diastolic blood pressure (F = 3.12, *p* = 0.039, R^2^ = 0.13) and fat mass (F = 4.39, *p* = 0.019, R^2^ = 0.15) compared to the ER and/or lTRE + ER groups, confirming that the follow-up cohort remained broadly representative of the randomized population.

During follow-up, only participants in the ER group significantly increased their energy intake compared to the end of the intervention period, leading to significant increases in body mass, body fat, and BMI. In contrast, participants in the TRE groups (eTRE + ER and lTRE + ER) significantly increased their eating window from the end of the intervention (on average, from 8.1 to 10.4 h in eTRE + ER and from 8.0 to 8.6 h in lTRE + ER), but their energy intake remained stable in both groups. Nevertheless, the difference in eating window between groups remained significant (*p* = 0.002). However, no significant differences were observed between groups in anthropometric and metabolic parameters during follow-up, suggesting similar trends across groups during this period (Table 1).

### 3.2. Impact of eTRE + ER, lTRE + ER, and ER Interventions on Gut Microbiome Diversity After a 3-Month Intervention and Follow-Up

**Figure 1:**
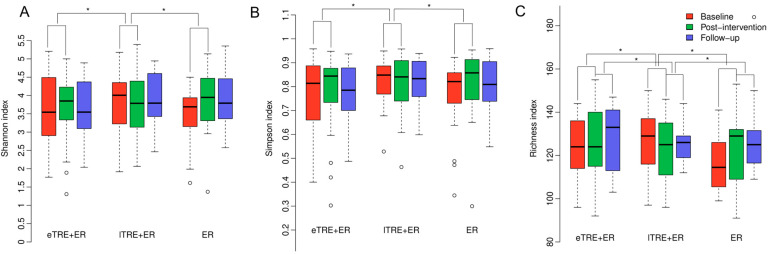
Microbiome alpha diversity at baseline (*n* = 76; eTRE + ER, *n* = 33; lTRE + ER, *n* = 23; ER, *n* = 20), at the end of the 3-month intervention (*n* = 76; eTRE + ER, *n* = 33; lTRE + ER, *n* = 23; ER, *n* = 20) and after follow-up (*n* = 43; eTRE + ER, *n* = 14; lTRE + ER, *n* = 14; ER, *n* = 15) in all three groups (eTRE + ER, lTRE + ER, ER). (**A**) Shannon index; (**B**) Simpson index; (**C**) Richness index. Values are medians and 95% CI. * Significant post hoc comparisons of changes between group, *p* < 0.05. ° Visible outliers.

To assess whether gut microbiota diversity and composition differed among participants undergoing different intervention protocols (eTRE + ER, lTRE + ER, ER), we analysed microbiome signatures before and after the 3-month intervention and again after a 6-month follow-up. No significant differences in beta diversity were observed during the interventions between groups (R^2^ = 0.216, *p* = 0.117), suggesting that a 3-month TRE+ER or ER treatment does not substantially alter the overall gut microbiome structure. On the other hand, there were significant differences between groups during intervention in alpha diversity, including Shannon index (F = 5.72, *p* < 0.001, R^2^ = 0.14), Simpson index (F = 6.72, *p* < 0.001, R^2^ = 0.14) (Figure 1A,B), and richness (F = 3.99, *p* = 0.01, R^2^ = 0.11, Figure 1C). Specifically, microbiome alpha diversity decreased (although not significantly) in the lTRE + ER group, whereas it remained stable or increased (not significantly) in the eTRE + ER group and in the ER group, respectively (Figure 1). During follow-up, we detected significant differences between groups in richness only (F = 3.77, *p* = 0.018, R^2^ = 0.09), with reductions in the ER group and increases in the TRE + ER (Figure 1). Otherwise, no additional significant changes were observed in beta or alpha diversity, including Shannon and Simpson indexes, suggesting that gut microbiota structure and diversity remained almost stable over the follow-up period.

### 3.3. Impact of eTRE + ER, lTRE + ER and ER Interventions on Gut Microbiome Composition at the Phylum and Genus Level After a 3-Month Intervention and During Follow-Up

The average microbiota community composition at phylum level is presented in Table 2. The two most common phyla were Bacillota and Bacteroidota, accounting for approximately 48.3% and 45.8%, respectively, of the total abundance at baseline. No significant between-group differences in gut microbiome composition at the phylum level were observed before and after the interventions in either the full sample (*n* = 76) or the follow-up sample (*n* = 43), or after follow-up (Table 2), confirming that a 3-month TRE+ER or ER treatment does not substantially alter the overall gut microbiome structure at the phylum level. On the other hand, some significant changes in gut microbiota relative abundances at the phylum level were detected within groups. At the end of the 3-month intervention, no significant shifts were detected within eTRE + ER and lTRE + ER groups, whereas significant increases in Verrucomicrobiota were found in the ER group (Table 2). This pattern was consistent in both the full and follow-up samples. Interestingly, during follow-up, significant increases in Bacteroidota and Desulfobacterota, and significant reductions in Bacillota were observed in the eTRE + ER group, while in lTRE + ER and ER groups only significant increases in Desulfobacterota were found (Table 2). Additionally, the significant reductions in ratio Bacillota/Bacteroidota were detected only in the eTRE + ER group during follow-up.

**Figure 2:**
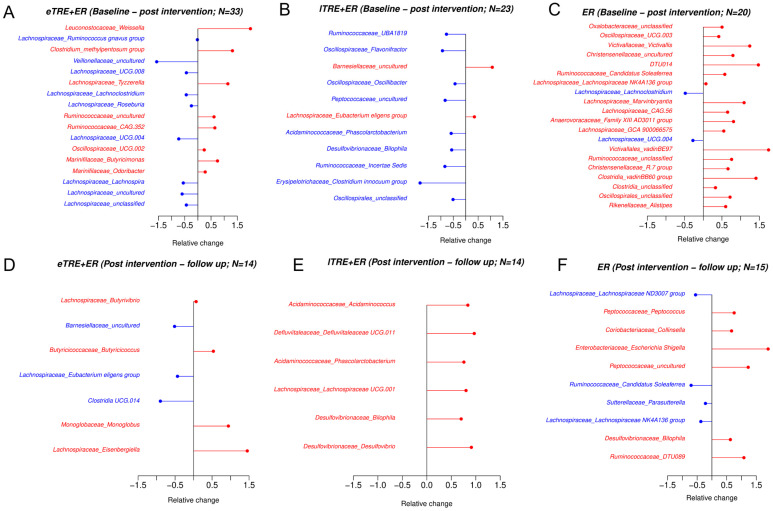
Significantly different abundances at the genus level within each group at the end of the intervention and after follow-up. Significantly different abundances at the genus level within eTRE + ER group, *n* = 33 (**A**), lTRE + ER group, *n* = 23 (**B**), and ER group, *n* = 20 (**C**), at the end of the intervention compared with the baseline values (relative changes are presented; significant reductions are presented in a blue colour, increases in red). Significantly different abundances at the genus level within eTRE + ER group, *n* = 14 (**D**), lTRE + ER, *n* = 14 (**E**), and ER group, *n* = 15 (**F**), during follow-up (relative changes are presented; significant reductions are presented in a blue colour, increases in red). Wilcoxon signed-rank tests were applied and *p* < 0.05 was used as significantly different.

Next, we used the Wilcoxon test to identify differential taxa at the genus level before and after the 3-month intervention, as well as during follow-up. In the eTRE + ER group, 17 genera showed significant changes, with 9 decreasing (all from the *Lachnospiraceae family*) and 8 increasing (Figure 2A). The lTRE + ER group exhibited 11 changed genera, with 9 decreasing and 2 increasing (Figure 2B), while the ER group showed 21 changes, with 2 genera decreasing and 19 increasing (Figure 2C). During follow-up, fewer differences were observed across all groups. In the eTRE + ER group, seven genera changed, with four decreasing and three increasing (Figure 2D). The lTRE + ER group displayed only increases in six genera (Figure 2E), whereas the ER group showed four genera decreasing and six increasing (Figure 2F).

**Figure 3:**
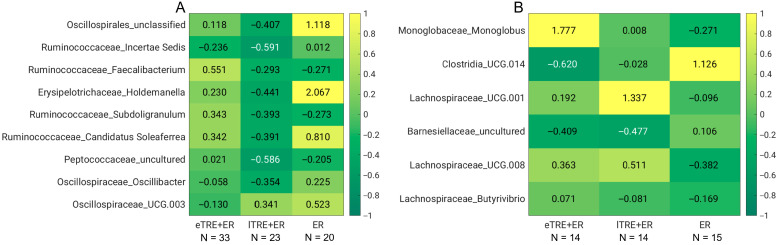
Significant differences in changes in genera abundances during intervention period (**A**) and follow-up (**B**) between groups. Wilcoxon-sum tests were applied and relative differences in each genus and each group after the intervention period and during follow-up are presented. Decreases are coloured in green and increases in yellow.

When analysing significant differences in genus-level abundance changes during the intervention period across groups, a total of 19 genera exhibited significant changes between groups. These included changes in *Oscillospirales_unclassified*, genera from *Erysipelotrichaceae* (*Holdemanella*, *Clostridium innocuum group*), *Ruminococcaceae* (*Incertae Sedis*, *Subdoligranulum*, *Faecalibacterium, Candidatus Soleaferrea*), *Oscillospiraceae* (*Oscillibacter*, *UCG.003*), and *Lachnospiraceae* (*Eisenbergiella*, *Marvinbryantia*, *GCA.900066755*), as well as *Peptococcaceae_uncultured*, *Atopobiaceae_Olsenella*, *Actinomycetaceae_Actinomyces*, *DTU014, Gemellaceae_Gemella*, *Leuconostocaceae_Weissella*, and *Hungateiclostridiaceae_Ruminiclostridium.* Among these, only 9 out of 19 genera were present in more than 50% of participants in each group (Figure 3A).

Changes in *Ruminococcaceae_Faecalibacterium* were significantly different between groups (*p* = 0.008), with increases observed in the eTRE + ER group and reductions in the lTRE + ER (*p* = 0.004 vs. eTRE + ER) and in the ER group (*p* = 0.021 vs. eTRE + ER). A similar trend was observed also for *Peptococcaceae_uncultured* (*p* = 0.032 between groups, *p* = 0.014 between eTRE + ER and lTRE + ER goups, and *p* = 0.045 between eTRE + ER and ER groups), and *Ruminococcaceae_Subdoligranulum* (*p* = 0.036 between groups and *p* = 0.007 between eTRE + ER and lTRE + ER goups). Additionally, changes in *Erysipelotrichaceae_Holdemanella, Oscillospirales_unclassified,* and *Oscillospiaceae_Oscillibacter* at the end of the intervention were significantly different between groups (*p* = 0.049, *p* = 0.002, and *p* = 0.034, respectively). Specifically, reductions in these genera in the lTRE + ER group were significantly different compared to the increases observed in the ER group (*p* = 0.020, *p* < 0.001, and *p* = 0.024, respectively). Moreover, changes in *Oscillospiaceae_UCG.003* also varied significantly between groups (*p* = 0.015), with reductions in eTRE + ER differing significantly from the increases in ER (*p* = 0.003). Furthermore, changes in *Ruminococcaceae* (*Incertae sedis* and *Candidatus soleaferrea*) were significantly different between groups at the end of the 3-month intervention (*p* = 0.049 and *p* = 0.012, respectively). Post hoc tests revealed significant differences between eTRE + ER and lTRE + ER (*p* = 0.039 and *p* = 0.021, respectively), and between lTRE + ER and ER groups (*p* = 0.025 and *p* = 0.003, respectively). In both cases, reductions in lTRE + ER were significantly different compared to the other two groups (Figure 3A).

During follow-up, changes in six genera, present in more than 50% of participants in each group, significantly differed between groups (Figure 3B). Changes in *Monoglobaceae_Monoglobus* were significantly different across groups (*p* = 0.018), with increases in the eTRE + ER group, reductions in the ER group (*p* = 0.009 between these groups) and no changes in the lTRE + ER group (*p* = 0.039 between eTRE + ER and lTRE + ER). Additionally, significant differences between groups were observed for *Clostridia UCG.014* and *Barnesiellaceae_uncultured* (*p* = 0.006 and *p* = 0.034, respectively). In both cases, reductions in the eTRE + ER group were significantly different from increases in the ER group (*p* = 0.002 and *p* = 0.008, respectively). Furthermore, *Clostridia UCG.014* changes differed significantly between lTRE + ER and ER (*p* = 0.046). Significant between-group differences were also observed for *Lachnospiraceae_Butyrivibrio* (*p* = 0.041) with increases in the eTRE + ER group and reductions in lTRE + ER (*p* = 0.041 vs. eTRE + ER) and in ER (*p* = 0.025 vs. eTRE + ER). Lastly, *Lachnospiraceae_Lachnospiraceae UCG.008* and *UCG.001* exhibited significant differences across groups during follow-up (*p* = 0.010 and *p* = 0.039, respectively), with reductions in the ER group and increases in both TRE groups, especially in lTRE + ER. Pairwise comparisons showed significant differences between eTRE + ER and ER (*p* = 0.034 and *p* = 0.031, respectively) and between lTRE + ER and ER (*p* = 0.006 and *p* = 0.029, respectively).

### 3.4. Associations of Taxonomic Alterations with Changes in Clinical Parameters Induced by Interventions

**Figure 4:**
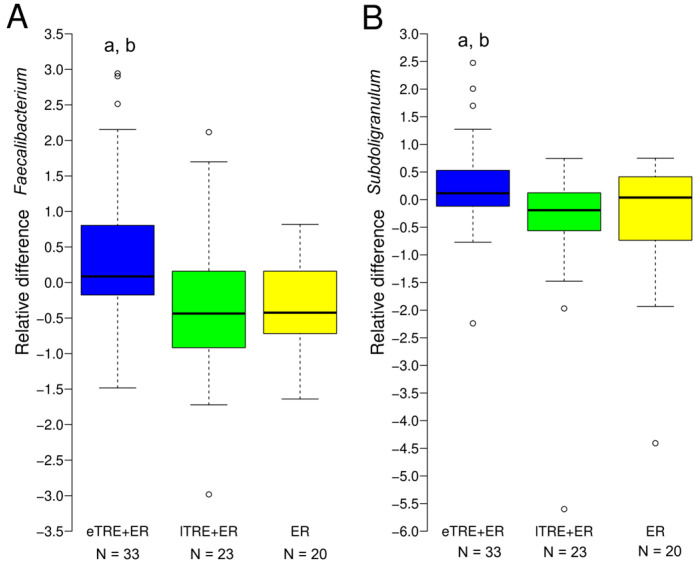
Changes in relative abundances during intervention period of *Faecalibacterium* (**A**) and *Subdoligranulum* (**B**). Changes in relative abundances and follow-up (**B**) between groups. Values are medians and 95% CI. ^a^ Significant post hoc comparison of changes between eTRE + ER (blue colour) and lTRE + ER (green colour). ^b^ Significant post hoc comparison of changes between eTRE + ER (blue colour) and ER (yellow colour), *p* < 0.05. ° Visible outliers.

Given the significant differences in changes in fasting glucose and DBP at the end of the interventions between the three intervention groups in our parent study [4] and in the present sample, and as interventions promoted divergent functional shifts in gut microbiome between groups, a Spearman correlation analysis was performed between differential taxa at the genus level and differential changes in fasting glucose and DBP between groups. Table 3 presents significant associations between changes in relative abundances of specific genera and changes in health parameters that differed between groups. Changes in *Faecalibacterium* were significantly negatively related to changes in fasting glucose. Specifically, *Faecalibacterium* increased in the eTRE + ER group (Figure 3A and Figure 4A), where fasting glucose was reduced to a greater extent, whereas a reduction in *Faecalibacterium* was observed in both the lTRE + ER and ER groups (Figure 3A and Figure 4A). Similarly, changes in *Subdoligranulum* were significantly negatively associated with changes in DBP. Indeed, *Subdoligranulum* increased in the eTRE + ER group (Figure 3A and Figure 4B), where DBP was reduced more significantly, whereas reductions in *Subdoligranulum* were detected in both the lTRE + ER and ER groups (Figure 3A and Figure 4B). Overall, although the mentioned associations were significant, they were weak and unadjusted for potential cofounders.

## 4. Discussion

In our parent study, we found that eTRE combined with ER was more effective in reducing fat mass percentage, DBP, and fasting glucose levels compared to lTRE with ER or ER alone after 12 weeks of intervention [4]. In comparison to the parent study, the sample here was smaller, due to the fact that for some participants faeces samples were not available. Nevertheless, the randomization was maintained in this secondary analysis, including the follow-up sample, and we confirmed the superior effects of eTRE + ER on fasting glucose and DBP compared to lTRE + ER and/or ER groups.

Given the gut microbiota’s role in energy homeostasis, nutrient absorption, appetite regulation, metabolic and inflammatory pathways, and circadian alignment, it is plausible that TRE could influence its composition and diversity. The human gut microbiota follows a circadian rhythm, and TRE—by reducing overall dietary intake and limiting nutrient availability to the distal colon during fasting—may help restore microbiome fluctuations disrupted by unhealthy lifestyle habits [14]. However, human studies on TRE and the gut microbiome remain limited and yield conflicting results [17,18,20,21,22,23,24,27]. Therefore, we conducted a secondary per-protocol analysis to determine whether adding TRE, particularly eTRE, to ER induces additional changes in gut microbiome composition compared to ER alone, and whether these changes are linked to metabolic health.

In this study, we did not observe significant changes in beta and alpha diversity of the gut microbiota post-TRE compared to pre-TRE. These findings align with those of Gabel et al. [23], Dawson et al. [24], Huang et al. [28], and Johnson et al. [27], where no significant microbiome diversity changes were noted, but differ from Zeb et al. [17] and Khan et al. [16], who reported increased microbial diversity following TRE. However, we found significant differences between groups at the end of the intervention; particularly, a reduction in Shannon, Simpson and richness indexes in the lTRE + ER group differed significantly in comparison to increases (although not significant) in ER and eTRE + ER, suggesting that lTRE + ER may be less favourable in regard to the gut microbiota diversity. The possibility that the changes in diversity may be due to different nutrient intake can be excluded, since the analysis of intakes revealed no differences between groups [4]. Although alpha diversity is often considered a general marker of microbial ecosystem health, small shifts may not necessarily translate into meaningful functional or clinical effects. After 6 months of follow-up, no significant within- or between-group differences were observed in beta and alpha diversity, including Shannon, and Simpson indexes, while significant changes were observed between groups in the richness index, with reductions in the ER group and increases in TRE + ER during the follow-up period, suggesting that ER may be less beneficial in relation to gut microbiota richness during follow-up when compared to TRE + ER. However, we noted that during follow-up, only participants in the ER group significantly increased their energy intake compared to the end of the intervention period, leading to significant increases in body mass, body fat, and BMI during follow-up.

Beyond diversity, previous studies have reported varying effects of TRE on gut microbiota composition, with some showing increased abundances of *Prevotellaceae, Bacteroidaceae,* and *Butyricicoccus pullicaecorum* [18,19,20], while others reported no significant changes [22,23,24]. In our study, at the end of the intervention period, the eTRE+ER group exhibited significant reductions in several genera from the *Lachnospiraceae* family *(Lachnospira, UCG.004, Roseburia, Lachnoclostridium, UCG.008, Ruminococcus gnavus group unclassified, uncultured*) and increases in genera from the *Marinifilaceae (Odoribacter, Butyricimonas*) and *Ruminococcaceae* families (*CAG.352, uncultured*). Although evidence from various studies suggests that *Lachnospiraceae* may support certain health functions, these genera have also been linked to metabolic syndrome, obesity, diabetes, liver diseases, and cardiovascular diseases [29]. High abundances of *Lachnospiraceae* have been positively correlated with elevated glucose and lipid serum metabolites, indicating metabolic disturbances [30,31,32]. Therefore, significant reductions in *Lachnospiraceae* could be related to better metabolic health observed after eTRE + ER intervention. Additionally, considering the diurnal rhythms of gut microbiota, where Bacteroidota and Verrucomicrobia peak during fasting and Bacillota peaks during feeding [14], TRE may contribute to reductions in Bacillota genera. However, the present study failed to confirm significant changes in gut microbiota composition at the phylum level at the end of the 3-month intervention. Thus, our findings somewhat contradict the general trends reported in studies involving energy restriction diets [33,34], but align with results from Gabel et al. [23] and Johnson et al. [27]. However, while no significant shifts in Bacillota, Bacteroidota, or Verrucomicrobia were detected at the end of the 3-month intervention, our study revealed significant reductions in specific genera belonging to Bacillota following eTRE + ER (nine vs. two genera following ER). More interestingly, during follow-up, although the eating window in the eTRE + ER group increased from an average of 8.0 to 10.4 h, Bacteroidota significantly increased, while Bacillota significantly decreased. These results suggest that eTRE + ER may confer additional long-term benefits, as a higher Bacillota/Bacteroidota ratio is associated with obesity [9,35]. However, we have to stress that phylum-level changes, including shifts in the Bacillota/Bacteroidota ratio, do not adequately capture the functional complexity of the gut microbiota. In the lTRE + ER group, consistent with decreases in alpha diversity indexes, several reductions in different genera were observed at the end of the 3-month intervention (nine reductions vs. two increases), while only increases in specific genera (six vs. no reductions) were significant during follow-up, indicating a restoration toward baseline gut microbiota composition and diversity. However, in both groups (lTRE + ER and ER), no significant shifts were observed at the phylum level.

Although eTRE + ER seems to be more beneficial in regard to gut microbiota composition, between-group analyses at the end of the intervention and during follow-up did not reveal any significant changes at the phylum level. However, the analyses revealed significant changes at the genus level. Specifically, significant between-group changes were detected in *Oscillospirales_unclassified, Erysipelotrichaceae_Holdemanella*, *genera from Ruminococcaceae* (*Incertae Sedis*, *Subdoligranulum*, *Faecalibacterium*, *Candidatus Soleaferrea*), and *Oscillospiraceae* (*Oscillibacter*, *UCG.003*), as well as *Peptococcaceae_uncultured*. In addition, changes in *Subdoligranulum* and *Faecalibacterium* significantly correlated, although weakly, with changes in health parameters that differed between groups. Notably, eTRE + ER was more effective in reducing fasting glucose compared to lTRE + ER and ER. In line with the significant greater reduction of fasting glucose levels were the increases in the genus *Faecalibacterium*. *Faecalibacterium* increased in the eTRE + ER group, but decreased in lTRE + ER and ER groups, and these changes were significantly different and associated with reductions in fasting glucose, additionally explaining why eTRE + ER was more effective in reducing fasting glucose than lTRE + ER or ER alone. It is known that *Faecalibacterium* is a key butyrate-producing bacterium known for its anti-inflammatory effects and beneficial role in gut homeostasis [36,37]. *Faecalibacterium* is often reduced in individuals with metabolic disorders, including obesity, insulin resistance, and type 2 diabetes mellitus, and several studies have reported negative correlations between *Faecalibacterium* abundance and fasting glucose and/or insulin levels [38,39,40]. This suggest that *Faecalibacterium* may mediate additional improvements in glucose metabolism in the eTRE + ER group. The significant greater abundance of *Faecalibacterium* after Ramadan fasting in comparison to the control group was observed in several human studies [17,18,41].

Similarly, eTRE + ER was more effective in reducing DBP compared to lTRE + ER and ER. Increases in *Subdoligranulum* in the eTRE + ER group differed significantly from the reductions observed in the other two groups, and a negative weak association between DBP and *Subdoligranulum* was confirmed. As an anti-inflammatory, butyrate-producing bacterium, *Subdoligranulum* has been found to be depleted in patients with coronary artery disease, and is probably implicated in blood pressure regulation via vascular function, inflammation reduction, and renin–angiotensin system modulation [42,43]. In line with our results regarding increases in *Faecalibacterium*, *Subdoligranulum*, and *Incertae Sedis* during eTRE + ER intervention, a previous study demonstrated that Ramadan-associated intermittent fasting was associated especially with an upregulation of *Ruminococcaceae* and butyric acid in a manner that correlates to improvement in human physiologic surrogate markers [41]. Moreover, Huang et al. [28] also demonstrated the significant relationship between *Ruminococcaceae_bacterium_D16* and changes in the body composition parameters, including whole body mass, BMI, waist circumference, body fat rate, and abdominal fat area. The microbiota-generated butyric acid, a highly bioactive compound, is known to promote metabolic benefits through the modulation of gut–brain neural circuits [44]. Although the present study did not confirm the superior effects of eTRE on fat mass loss without adjustments for gender, age and baseline values, the trend was seen, and the between-group changes in the genera *Ruminococcaceae Incertae Sedis* may be linked with reductions of fat mass percentage. These bacteria play a role in fermenting dietary fibre and facilitate the production of SCFAs, which contribute to gut health and metabolic benefits, including anti-inflammatory properties, and the regulation of blood glucose and cholesterol levels [45]. Additionally, increased levels of *Ruminococcaceae Incertae Sedis* have been associated with lower body fat and improved metabolic profiles [46]. We have to stress that weak-to-moderate correlations between SCFA-producing genera and metabolic markers are commonly reported in the literature, given the multifactorial nature of metabolic regulation. These genera (*Faecalibacterium*, *Subdoligranulum*, and *Ruminococcaceae Incertae Sedis*) may, therefore, contribute to—but not drive per se—metabolic outcomes.

Despite the valuable insights gained, our study has several limitations. First, faecal samples may not fully capture microbiota changes in the upper gastrointestinal tract. Recent studies [47,48] have shown that microbial composition and function can vary substantially along the gastrointestinal tract, with the small intestine often harbouring faster-growing and more metabolically active bacteria. These regional differences could mean that the faecal microbiome provides only a partial snapshot of the diet-induced microbial dynamics, particularly those related to nutrient absorption, circadian rhythms, and metabolic signalling. Future studies should incorporate samples from proximal gut regions for a more comprehensive analysis. Second, our sample size was determined based on primary outcomes related to body mass in the parent study [4], potentially limiting statistical power and amplifying individual variability. Therefore, the exploratory nature of our microbiota analysis, combined with the reduced sample size, limits the ability to detect small or subtle differences in microbial composition. Additionally, the present sample was smaller in comparison to the sample of the parent study; therefore, all results of the parent study were not confirmed without adjustments for age, gender and baseline values. Furthermore, BIA was used in the present study. While BIA is a clinically applicable method suitable for repeated measurements, its precision can vary, due to its sensitivity to hydration status and other physiological factors [49]. However, the controlled trial design and maintained randomization minimized confounding factors. Moreover, while our intervention lasted only 12 weeks, limiting long-term insights, the extended follow-up period provided additional assessment. Furthermore, we did not measure microbial metabolites at baseline, post-intervention, and during follow-up. Metabolites such as butyrate, acetate, and propionate, as well, may play an important role in the effect of the microbiome on the aetiology of obesity [9]. Future studies should also include measurements of microbial metabolites, which would provide valuable mechanistic insight into how taxonomic shifts may translate into metabolic improvements. Additionally, future studies should also incorporate functional and temporal analyses to better elucidate the biological pathways underlying the observed associations. Lastly, results are based on relative abundance, which may obscure true directional changes in bacterial load.

## 5. Conclusions

In conclusion, the present study highlights the intricate relationship between TRE, gut microbiota, and metabolic health. Our findings suggest that lTRE + ER may be less effective in maintaining or enhancing gut microbiota diversity compared to eTRE + ER and ER. Additionally, eTRE + ER appears to be more beneficial long-term and in promoting gut microbes associated with metabolic health—such as *Subdoligranulum*, and *Faecalibacterium*—which may contribute to reductions in fasting glucose and DBP. However, while associations were observed, they are modest in strength, unadjusted for different cofounders, and do not imply causality. On the other hand, between-group analyses at the end of the intervention and during follow-up did not reveal any significant changes at the phylum level or in beta diversity. Moreover, further research is needed to clarify the mechanisms underlying these associations and to explore the therapeutic potential of personalized TRE strategies for obesity management. Future studies should also examine microbial populations in the upper gastrointestinal tract and potential intestinal tissue remodelling, to enhance our understanding of the gut microbiome’s role in metabolic regulation.

## Figures and Tables

**Table 1:** Characteristics of study participants at baseline, post-intervention ^1^ and during follow-up ^2^.

Characteristics	eTRE + ER (*n* = 33, 14 ^#^)	lTRE + ER (*n* = 23, 14 ^#^)	ER (*n* = 20, 15 ^#^)	*p*-Value
Sex (F/M)	8/25, 5/9 ^#^	10/13, 5/9 ^#^	10/10, 7/8 ^#^	0.124/0.783 ^#^
Age (years)	44.3 ± 8.7	49.2 ± 8.7	48.4 ± 6.4	0.110
Baseline body mass (kg)	84.7 ± 12.7	87.6 ± 9.9	86.7 ± 15.6	0.517
^1^ Body mass change (kg)	−6.5 ± 2.5 ***	−6.0 ± 2.8 ***	−5.5 ± 2.9 ***	0.478
^2^ Body mass follow-up change (kg)	1.3 ± 2.8	1.2 ± 2.9	1.5 ± 2.3 *	0.978
Baseline BMI (kg/m^2^)	29.0 ± 3.0	28.8 ± 2.0	29.1 ± 3.8	0.845
^1^ BMI change (kg/m^2^)	−2.2 ± 1.0 ***	−2.0 ± 1.0 ***	−1.8 ± 1.0 ***	0.300
^2^ BMI follow-up change (kg/m^2^)	0.4 ± 0.8	0.4 ± 0.9	0.5 ± 0.8 *	0.995
Baseline body fat mass (%)	34.1 ± 6.4	31.8 ± 7.1	30.4 ± 6.9	0.096
^1^ Body fat mass change (%)	−3.5 ± 2.4 ***	−2.5 ± 1.7 ***	−2.7 ± 1.6 ***	0.142
^2^ Body fat mass follow-up change (%)	0.6 ± 2.8	0.6 ± 1.8	0.9 ± 1.5 *	0.840
Baseline muscle mass (kg)	53.1 ± 10.7	57.0 ± 10.8	57.4 ± 11.6	0.328
^1^ Muscle mass change (kg)	−1.5 ± 1.1 ***	−1.9 ± 1.2 ***	−1.7 ± 1.4 ***	0.382
^2^ Muscle mass follow-up change (kg)	0.4 ± 1.3	0.1 ± 1.0	0.3 ± 1.1	0.793
REE (kcal/day)	1570 ± 370	1680 ± 360	1650 ± 360	0.510
^1^ REE change (kcal/day)	−160 ± 290 **	−120 ± 230 *	−130 ± 200 **	0.844
^2^ REE follow-up change (kcal/day)	−60 ± 320	−90 ± 230	100 ± 180	0.124
Baseline SBP (mmHg)	134 ± 16	133 ± 10	131 ± 13	0.662
^1^ SBP change (mmHg)	−11 ± 12 ***	−10 ± 14 **	−9 ± 13 **	0.782
^2^ SBP follow-up change (mmHg)	1 ± 13	2 ± 13	4 ± 11	0.654
Baseline DBP (mmHg)	88 ± 9	85 ± 9	83 ± 10	0.130
^1^ DBP change (mmHg)	−12 ± 8 ***	−10 ± 9 ***	−6 ± 6 ***	0.031 ^b^
^2^ DBP follow-up change (mmHg)	4 ± 7 *	2 ± 9	1 ± 8	0.377
Baseline fasting glucose (mmol/L)	5.16 ± 0.55	5.41 ± 0.47	5.16 ± 0.69	0.249
^1^ Glucose change (mmol/L)	−0.41 ± 0.55 ***	−0.14 ± 0.30	−0.10 ± 0.55	0.047 ^a,b^
^2^ Glucose follow-up change (mmol/L)	0.19 ± 0.40	0.05 ± 0.37	0.22 ± 0.50	0.582
Baseline total cholesterol (mmol/L)	5.09 ± 1.03	5.86 ± 1.14	5.13 ± 0.85	0.019 ^a^
^1^ Total cholesterol change (mmol/L)	−0.21 ± 0.58 *	−0.24 ± 0.58 *	−0.24 ± 0.55 *	0.938
^2^ Total cholesterol follow-up (mmol/L) change	0.10 ± 0.56	−0.05 ± 0.74	−0.25 ± 0.65	0.369
Baseline triacylglycerols (mmol/L)	1.31 ± 1.45	1.67 ± 1.67	1.29 ± 0.63	0.209
^1^ Triacylglycerol change (mmol/L)	−0.24 ± 0.80 *	−0.27 ± 0.53 *	−0.08 ± 0.31	0.916
^2^ Triacylglycerol follow-up change (mmol/L)	0.11 ± 0.39	−0.22 ± 0.71	−0.01 ± 0.40	0.236
Baseline energy intake (kcal)	2320 ± 480	2360 ± 910	2380 ± 530	0.638
^1^ Energy intake change (kcal)	−650 ± 480 ***	−580 ± 650 ***	−700 ± 300 ***	0.378
^2^ Energy intake follow-up change (kcal)	50 ± 380	20 ± 280	280 ± 430 *	0.134
Baseline eating window (h)	12.9 ± 1.0	12.6 ± 1.1	12.7 ± 1.1	0.619
^1^ Eating window at week 12 (h)	8.1 ± 0.5 ***	8.0 ± 0.3 ***	12.1 ± 0.2	<0.001 ^b,c^
^2^ Eating window at follow-up (h)	10.4 ± 1.9 **	8.6 ± 0.9 *	12.1 ± 0.9	0.002 ^a,b,c^

BMI, body mass index; DBP, diastolic blood pressure; ER, energy restriction; eTRE, early time-restricted eating; lTRE, late time-restricted eating, REE, resting energy expenditure; SBP, systolic blood pressure. ^#^ Number of participants after follow-up: eTRE + ER (*n* = 14; 5M/9F), lTRE + ER (*n* = 14; 5M/9F) and ER (*n* = 15; 7M/8F). Differences in baseline characteristics between three groups were compared using ANOVA or Kruskal–Wallis test. ^1^ Changes in parameters during interventions were calculated as differences between values at the end of the intervention and values at baseline. Differences between groups during the intervention were analysed using general linear model (*p* values have been adjusted by the Bonferroni corrections for multiple test). ^2^ Follow-up changes were calculated as differences in each parameter between follow-up and the end of the intervention. Differences between groups during follow-up were analysed using general linear model (*p* values has been adjusted by the Bonferroni corrections for multiple test). ^a^ eTRE + ER vs. lTRE + ER; ^b^ eTRE + ER vs. ER; ^c^ lTRE + ER vs. ER. * Significant differences within each group (paired *t*-test or Wilcoxon test): *** *p* < 0.001, ** *p* < 0.01, * *p* < 0.05.

**Table 2:** Gut microbiota (phylum level) at baseline, post-intervention ^1^ and during follow-up ^2^.

Gut Microbiota at the Phylum Level	eTRE + ER (*n* = 33, 14 ^#^)	lTRE + ER (*n* = 23, 14 ^#^)	ER (*n* = 20, 15 ^#^)	*p*-Value
Baseline Bacteroidota (%)	49.7 ± 20.8	43.5 ± 18.7	44.3 ± 23.2	0.413
^1^ Bacteroidota after 3-month change (%)	−4.4 ± 20.9	5.0 ± 18.6	0.6 ± 17.0	0.201
^2^ Bacteroidota follow-up change (%)	10.2 ± 13.2 *	1.0 ± 23.0	3.0 ± 16.8	0.377
Baseline Bacillota (%)	43.1 ± 19.9	51.4 ± 20.4	50.3 ± 24.0	0.241
^1^ Bacillota after 3-month change (%)	4.7 ± 21.7	−6.6 ± 20.9	−1.0 ± 18.7	0.123
^2^ Bacillota follow-up change (%)	−8.5 ± 13.4 *	−1.9 ± 24.5	−4.9 ± 14.6	0.632
Baseline Desulfobacterota (%)	0.4 ± 0.8	0.3 ± 0.5	0.2 ± 0.4	0.431
^1^ Desulfobacterota after 3-month change (%)	−0.1 ± 0.8	−0.1 ± 0.3	0.0 ± 0.3	0.475
^2^ Desulfobacterota follow-up change (%)	0.1 ± 0.2 *	0.2 ± 0.4 *	0.1 ± 0.3 *	0.948
Baseline Proteobacteria (%)	4.9 ± 6.6	3.7 ± 5.1	3.8 ± 5.1	0.503
^1^ Proteobacteria after 3-month change (%)	−0.4 ± 6.7	0.6 ± 8.0	−0.5 ± 5.1	0.595
^2^ Proteobacteria follow-up change (%)	−1.1 ± 5.2	1.3 ± 4.4	3.0 ± 6.8	0.433
Baseline Cyanobacteria (%)	0.5 ± 1.4	0.3 ± 0.7	0.5 ± 1.4	0.953
^1^ Cyanobacteria after 3-month change (%)	0.5 ± 2.7	0.6 ± 3.0	0.5 ± 2.9	0.920
^2^ Cyanobacteria follow-up change (%)	−0.7 ± 2.5	−0.6 ± 4.3	−0.6 ± 2.7	0.556
Baseline Actinobacteriota (%)	0.4 ± 0.5	0.4 ± 0.7	0.4 ± 0.6	0.578
^1^ Actinobacteriota after 3-month change (%)	0.3 ± 1.1	−0.2 ± 0.8	−0.1 ± 0.5	0.297
^2^ Actinobacteriota follow-up change (%)	−0.2 ± 0.6	−0.1 ± 0.6	0.0 ± 0.4	0.459
Baseline Verrucomicrobiota (%)	1.0 ± 2.4	0.3 ± 0.5	0.2 ± 0.4	0.072
^1^ Verrucomicrobiota after 3-month change (%)	−0.4 ± 2.3	0.6 ± 2.2	0.5 ± 1.3 *	0.541
^2^ Verrucomicrobiota follow-up change (%)	0.3 ± 1.8	0.0 ± 0.5	−0.4 ± 1.3	0.966
Baseline Bacillota/Bacteroidota ratio	1.5 ± 1.6	3.4 ± 8.4	2.1 ± 2.3	0.336
^1^ Bacillota/Bacteroidota ratio after 3-month change	0.2 ± 1.5	−2.0 ± 7.6	−0.7 ± 1.9	0.188
^2^ Bacillota/Bacteroidota ratio follow-up change	−1.1 ± 1.8 *	−0.1 ± 1.0	−0.1 ± 0.8	0.071

ER, energy restriction; eTRE, early time-restricted eating; lTRE, late time-restricted eating. ^#^ Number of participants after follow-up: eTRE + ER (*n* = 14; 5M/9F), lTRE + ER (*n* = 14; 5M/9F) and ER (*n* = 15; 7M/8F). Differences in baseline characteristics between three groups were compared using ANOVA or Kruskal–Wallis test. ^1^ Changes in parameters during interventions were calculated as differences between parameters at the end of the intervention and parameters at baseline. ^2^ Follow-up changes were calculated as differences in each parameter between follow-up and the end of the intervention. * Significant differences within each group (paired *t*-test or Wilcoxon test): * *p* < 0.05.

**Table 3:** Significant associations of taxonomic alterations with changes in clinical parameters induced by interventions.

Changes in Relative Abundances—Different Between Groups (Genera)	Changes in Metabolic and Anthropometric Parameters—Different Between Groups	
	∆ Glucose (mmol/L)	∆ DBP (mmHg)
∆ *Faecalibacterium*	−0.226 *	−0.029
∆ *Subdoligranulum*	0.056	−0.238 *

DBP, diastolic blood pressure. Correlation analysis between changes in health markers—significantly different between groups at the end of the 3-month intervention and relative taxonomic alterations caused by a 3-month intervention. * *p* < 0.05. Only changes in genera—different between groups and with significant correlations with changes in health markers are shown.

## Data Availability

Data from this article will be made available upon reasonable request from the corresponding author. The data are not publicly available due to ongoing study.

## Abbreviations

eTRE	Early time-restricted eating
lTRE	Late time-restricted eating
HDL	High-density lipoprotein
LDL	Low-density lipoprotein
TG	Triacylglycerols
DBP	Diastolic blood pressure
TRE	Time-restricted eating
T2DM	Type 2 diabetes mellitus
SCFAs	Short-chain fatty acids
ER	Energy restriction
SD	Standard deviation
